# Integrated strategy combining endobronchial ultrasound with positron emission tomography to diagnose peripheral pulmonary lesions

**DOI:** 10.1111/1759-7714.13484

**Published:** 2020-06-16

**Authors:** Xiao Wu, Zhou An, Kui Zhao, Sijia Yang, Xu Lin, Xiaona Dai, Derek Radisky, Jian Hu

**Affiliations:** ^1^ Department of Thoracic Surgery First Affiliated Hospital, College of Medicine, Zhejiang University Hangzhou China; ^2^ PET Center First Affiliated Hospital, College of Medicine, Zhejiang University Hangzhou China; ^3^ Hospital Administration Office of Zhejiang University Hangzhou China; ^4^ Department of Cancer Biology Mayo Clinic Cancer Center Jacksonville Florida USA

**Keywords:** Diagnosis, endobronchial ultrasound, peripheral pulmonary lesions, positron emission tomography, transbronchial lung biopsy

## Abstract

**Background:**

Endobronchial ultrasound‐guided transbronchial lung biopsy (EBUS‐TBLB) and fluorodeoxyglucose positron emission tomography (FDG‐PET) have been widely used in the diagnosis of peripheral pulmonary lesions (PPLs). This study was conducted to determine the diagnostic value of EBUS‐TBLB combined with FDG‐PET in the assessment of PPLs.

**Methods:**

The clinical data of 76 patients with PPLs who received both FDG‐PET and EBUS‐TBLB from January 2016 to February 2018 were retrospectively evaluated. Further subgroup analysis was performed according to lesion diameter (≤20 mm or >20 mm). Related diagnostic indices were calculated and compared between groups.

**Results:**

When combining EBUS‐TBLB with FDG‐PET, the diagnostic accuracy rate, sensitivity, specificity, Youden's index, positive predictive value, and negative predictive value for PPLs were 86.8%, 90.2%, 73.3%, 63.5%, 93.2%, and 64.7%, respectively. In addition, the diagnostic accuracy rate of the combined approach was significantly higher than the single EBUS‐TBLB and FDG‐PET (*P* < 0.01 and *P* < 0.05, respectively), and its Youden's index was also at a higher level. When stratified by lesion diameter, the combined approach showed a significantly higher diagnostic accuracy rate (*P* < 0.05) and a higher Youden's index for PPLs >20 mm than PPLs ≤20 mm. In addition, we found that positive bronchus sign and probe within the probe were two important factors conducing to enhancing the diagnostic accuracy rate for EBUS‐TBLB.

**Conclusions:**

An integrated approach combining EBUS‐TBLB with FDG‐PET is particularly useful for diagnosing PPLs, and the improved diagnostic yields were especially evident for PPLs >20 mm.

## Introduction

Lung cancer is one of the most frequently diagnosed malignancies on a global scale. ^1^ With the popularization of low‐dose computed tomography (LDCT) screening, the frequency of incidentally discovered peripheral pulmonary lesions (PPLs) has increased greatly.^2^ In addition, a recent meta‐analysis revealed a high median prevalence of malignancy among PPLs, 68% (range 50%–84%).^3^ Therefore, it is imperative to identify malignant PPLs at their earliest stages.

A variety of medical techniques have been adopted to diagnose PPLs. Among these techniques, fluorodeoxyglucose positron emission tomography (FDG‐PET) is an important approach to discriminate between malignant and benign PPLs. As for FDG‐PET, maximum standardized uptake value (SUV_max_) is a critical index for evaluating FDG uptake, and SUV_max_ >2.5 is generally regarded as a cutoff value for diagnosing malignant lesions.^4^, ^5^, ^6^, ^7^, ^8^ To be consistent, in our study, the traditional cutoff SUV_max_ of 2.5 was adopted for distinguishing malignant from benign PPLs. According to the findings of Mizugaki *et al*. FDG‐PET has a diagnostic accuracy rate of 78.5% for PPLs.^9^ Thus, there is a need to resort to biopsy to make a definitive diagnosis.

Due to the superior imaging guidance of endobronchial ultrasound‐guided transbronchial lung biopsy (EBUS‐TBLB), this has been widely used in the diagnosis of PPLs.^10^, ^11^ According to the clinical practice guidelines of the American College of Chest Physicians, radial EBUS is recommended as an auxiliary imaging modality for diagnosing PPLs.^12^ Since Kurimoto *et al*.^13^ first reported that endobronchial ultrasonography using a guide sheath (EBUS‐GS) could be used to increase the ability to diagnose PPLs, several studies have confirmed its safety and efficacy.^11^, ^14^, ^15^ Furthermore, Mizugaki H *et al*. have found an integrated approach combining EBUS‐GS with FDG‐PET could increase the diagnostic yields for PPLs ≤30 mm.^9^ Despite the superiority of EBUS‐GS for diagnosing PPLs, the diagnostic accuracy rate of EBUS‐TBLB without GS could also reach 66.7%–77.0%.^16^, ^17^ In addition, the adoption of GS (a disposable supply) can significantly increase patients' operational expenses, and enlarge the caliber of the ultrasonic probe which may hinder the operation into the small angled branch of peripheral bronchi.^17^, ^18^ Allowing for the above defects of EBUS‐GS, EBUS‐TBLB without GS is still employed at some hospitals especially in bronchoscopy units without GS. Moreover, a recent meta‐analysis reported that a multimodality approach might be more conducive to diagnosing peripheral lesions.^19^ Therefore, an integrated strategy may be more preferable to evaluate PPLs.

To our knowledge, there are few studies regarding an integrated approach which combines EBUS‐TBLB with FDG‐PET for diagnosing PPLs. This study was conducted to determine the role of EBUS‐TBLB combined with FDG‐PET in the assessment of PPLs.

## Methods

### Patients

From 1 January 2016 to 28 February 2018, 76 consecutive patients with PPLs who received EBUS‐TBLB at the First Affiliated Hospital of Zhejiang University and FDG‐PET at tertiary care centers were enrolled in this retrospective study. PPLs were generally defined as lesions encircled by normal lung parenchyma without showing any evidence of endobronchial abnormalities. EBUS‐TBLB was performed within four weeks after FDG‐PET was examined. Whether PPLs were malignant or not was ultimately confirmed by the histological diagnosis based on the invasive procedures (such as percutaneous needle biopsy and surgical operations), or imaging and clinical follow‐up examinations. All PPLs were divided into two groups on the basis of lesion diameter: ≤20 mm and >20 mm. This study was approved by the ethics committee at the First Affiliated Hospital of Zhejiang University. All patients provided their written informed consent.

### EBUS‐TBLB

Bronchoscopy was carried out with a flexible bronchoscope (BF‐1T260; Olympus) under local anesthesia. For patients receiving EBUS‐TBLB, ultrasound analysis was carried out employing an EBUS system (processor EU‐ME2; Olympus), equipped with a 20 MHz mechanical radial miniprobe (UM‐S20‐20R; Olympus). A thorough examination of the target bronchus with the bronchoscope was performed. Once the target PPL was localized, we measured the distance between it and the outer orifice of the bronchoscope working channel for the purpose of performing TBLB at the same PPL site. Subsequently, the miniprobe was withdrawn, and biopsy forceps were inserted into the bronchoscope working channel to acquire five or more biopsy specimens in the same location and depth indicated by the minprobe.^20^ In addition, bronchial brushes were introduced routinely to acquire the cytological specimens. The final diagnosis of EBUS‐TBLB was based on the pathological and cytological specimens. All procedures were performed by experienced thoracic surgeons who possessed at least five years of experience in bronchoscopy.

### FDG‐PET

FDG‐PET scan was performed using an integrated FDG‐PET scanner (Biograph Sensation 16, LSO 39 ring, Siemens Medical, Erlangen, Germany). All patients were informed to fast over six hours (blood glucose <8 mmol/L) prior to the examination and were subsequently intravenously injected with FDG at a level of 5.5–7.4 MBq/kg bodyweight. After resting for an hour, patients received a whole‐body FDG‐PET scan. Tumor/background (T/B) ratio was utilized to determine the tumor uptake of ^18^F‐FDG. Regions of interest (ROIs) were drawn over the lesions with the highest uptake of ^18^F‐FDG, and then the SUV_max_ was calculated automatically by the computer. In this study, SUV_max_ >2.5 was regarded as a cutoff value for the diagnosis of malignant lesions, which was consistent with previous studies.^4^, ^5^, ^6^, ^7^, ^8^

### Statistical analysis

Categorical variables were reported as median (interquartile range, IQR). Diagnostic indices including diagnostic accuracy rate, sensitivity, specificity, Youden's index, positive predictive value and negative predictive value were calculated, respectively. In addition, we also calculated the diagnostic accuracy rate of different clinical characteristics, such as age, bronchus sign on CT, and probe position. These characteristics are influencing factors, either for EBUS‐TBLB or PET.^21^, ^22^, ^23^, ^24^ Comparisons among different diagnostic accuracy rates were performed using McNemar test for paired data and chi‐squared test (with or without continuity correction) for unpaired data. A nonparametric Kruskal‐Wallis test was used to compare the SUV_max_ of different subgroups. All statistical analyses were performed using R version 3.5.1 software. A two‐tailed *P*‐value < 0.05 was regarded as statistically significant.

## Results

Baseline information of the eligible patients is described in Table 1. In general, the data of 76 patients with PPLs were analyzed in this study. Patients' median age was 64 (IQR, 58–70) years and the proportion of males to females was 62% to 38%. A total of 48 (63.1%) patients were observed with bronchus sign on CT, and 51 (67.1%) probes were within the PPLs. The final diagnoses of 76 patients are presented in Table 2. In total, 70 patients were confirmed by invasive procedures, while six patients were confirmed by imaging and clinical follow‐up examinations. In addition, a total of 61 patients were confirmed to have malignant PPLs, of which 38 were identified by EBUS‐TBLB. Complications resulting from EBUS‐TBLB or FDG‐PET that required hospitalization did not occur in this study.

**Table 1:** Characteristics of patients with peripheral pulmonary lesions (PPLs) (*n* = 76)

Characteristics	Distribution
Age (years)	
Median (IQR)	64 (58–70)
Gender	
Male	47 (62%)
Female	29 (38%)
Lesion size (mm)	
Median (IQR)	28 mm (20–39 mm)
Tumor location	
Left upper lobe	13 (17.1%)
Left lower lobe	14 (18.4%)
Right upper lobe	23 (30.3%)
Right middle lobe	10 (13.2%)
Right lower lobe	16 (21.1%)
CT bronchus sign	
Positive	48 (63.1%)
Negative	28 (36.9%)
Probe within the lesions	
Yes	51 (67.1%)
No	25 (32.9%)
Pleural effusion	
Yes	8 (10.5%)
No	68 (89.5%)
Disease history	
Diabetes mellitus	8 (10.5%)
Tuberculosis	3 (3.9%)
Malignant tumors	5 (6.6%)
SUV_max_	
Median (IQR)	7.1 (2.3–9.8)

IQR, interquartile range; PPLs, peripheral pulmonary lesions; SUV_max_, maximum standardized uptake value.

**Table 2:** Final diagnosis of 76 patients in our study

	Patients	Diagnosed by EBUS	Diagnosed by PET‐CT
Final diagnosis	No	No (All)	No (All)
Malignant	61	38(61)	48(61)
Lung cancer	58	36(58)	
Adenocarcinoma	41	25(41)	
Squamous cell carcinoma	5	3(5)	
Small cell carcinoma	5	4(5)	
Non‐small cell carcinoma	7	4(7)	
Metastatic lung cancer	2	1(2)	
Lymphoma	1	1(1)	
Benign	15	7(15)	11(15)
Pneumonia	10	6(10)	
Tuberculosis	2	1(2)	
Others	3	0(3)	

EBUS, endobronchial ultrasonography.

### Diagnostic value of EBUS‐TBLB for PPLs

All lesions were detected and punctured by EBUS‐TBLB. The value of EBUS‐TBLB in the diagnosis of PPLs was presented with a diagnostic accuracy rate of 69.7%, sensitivity of 62.3%, specificity of 100%, Youden's index of 62.3%, positive predictive value of 100%, and negative predictive value of 39.5% (Table 3). When compared with PPLs ≤20 mm, EBUS‐TBLB for PPLs >20 mm showed a significantly higher diagnostic accuracy rate (*P* < 0.05) and a higher Youden's index.

**Table 3:** The diagnostic indices of the single method and combined method

	Diagnostic indices					
Examination	Accuracy rate (%) (95% CI)	Sensitivity (%) (95% CI)	Specificity (%) (95% CI)	Youden's index (%) (95% CI)	Positive predictive value (%) (95% CI)	Negative predictive value (%) (95% CI)
EBUS‐TBLB	69.7(58.1–79.8)**	62.3(49.0–74.4)	100(78.2–100)	62.3(27.2–74.4)	100(90.7–100)	39.5(24.0–56.6)
PPLs ≤20 mm	52.4(29.8–74.3)*,***	37.5(15.2–64.6)****	100(47.8–100)	37.5(−37.0–64.6)	100(54.1–100)	33.3(11.8–61.6)
PPLs >20 mm	76.4(63.0–86.8)**	71.1(55.7–83.6)	100(69.2–100)	71.1(24.8–83.6)	100(89.1–100)	43.4(23.2–65.5)
PET	77.6(66.6–86.4)*	78.7(66.3–88.1)	73.3(44.9–92.2)	52.0(11.2–80.3)	92.3(81.5–97.9)	45.8(25.6–67.2)
PPLs ≤20 mm	61.9(38.4–81.9)***	62.5(35.4–84.8)	60.0(14.7–94.7)	22.5(−49.9–79.5)	83.3(51.6–97.9)	33.3(7.5–70.1)
PPLs >20 mm	83.6(71.2–92.2)	84.4(70.5–93.5)	80.0(44.4–97.5)	64.4(14.9–91.0)	95.0(83.1–99.4)	53.3(26.6–78.7)
EBUS‐TBLB+PET	86.8(77.1–93.5)	90.2(79.8–96.3)	73.3(44.9–92.2)	63.5(24.7–88.5)	93.2(83.5–98.1)	64.7(38.3–85.8)
PPLs ≤20 mm	71.4(47.8–88.7)***	75.0(47.6–92.7)	60.0(14.7–94.7)	35.0(−37.7–87.5)	85.7(57.2–98.2)	42.9(9.9–81.6)
PPLs >20mm	92.7(82.4–98.0)	95.6(84.9–99.5)	80.0(44.4–97.5)	75.6(29.2–96.9)	95.6(84.9–99.5)	80.0(44.4–97.5)

Note: *P < 0.05 compared to EBUS‐TBLB+PET; **P < 0.01 compared to EBUS‐TBLB+PET; ***P < 0.05 compared to PPLs >20 mm; ****P < 0.01 compared to PPLs >20 mm.

CI, confidence interval.

### Diagnostic value of FDG‐PET for PPLs

**Figure 1:**
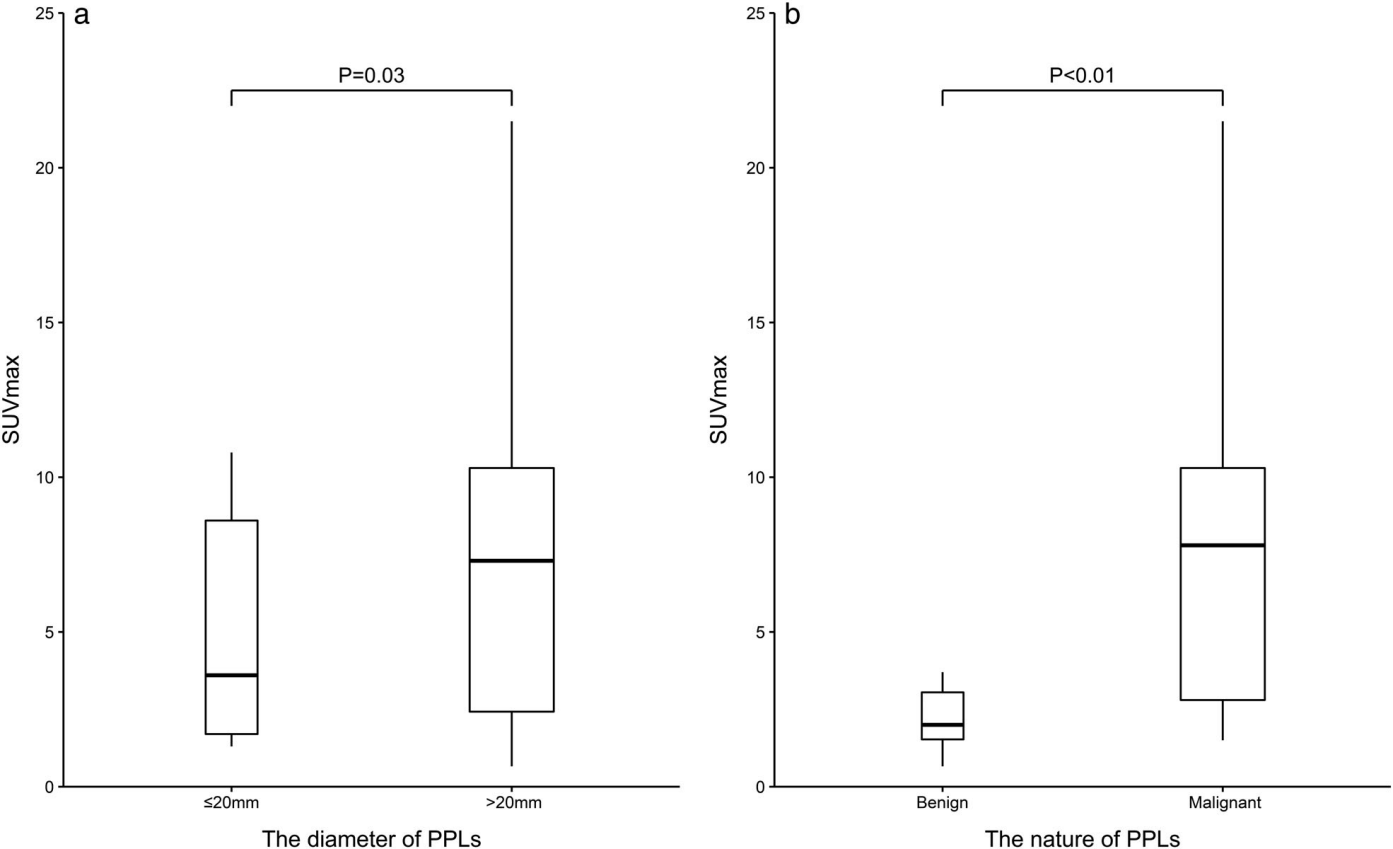
The SUV_max_ of FDG‐PET for PPLs grouped by the lesion diameter and malignancy. Note: boxes are drawn with widths proportional to the square‐root of the number of observations in the groups. IQR, interquartile range; PPLs, peripheral pulmonary lesions; SUV_max_, maximum standardized uptake value; FDG‐PET, fluorodeoxyglucose positron emission tomography.

The median SUV_max_ of FDG‐PET was 2.0 (IQR, 1.5–3.1) for benign PPLs, 7.8 (2.8–10.3) for malignant PPLs, 3.6 (1.7–8.6) for PPLs ≤20 mm, and 7.3 (2.4–10.3) for PPLs >20 mm (Table 4, Fig 1). Significantly higher levels of SUV_max_ were found in malignant PPLs and PPLs >20 mm (*P* < 0.01 and *P* = 0.03, respectively) (Table 4, Fig 1). Meanwhile, the diagnostic value of FDG‐PET in the diagnosis of PPLs was presented with a diagnostic accuracy rate of 77.6%, sensitivity of 78.7%, specificity of 73.3%, Youden's index of 52.0%, positive predictive value of 92.3%, and negative predictive value of 45.8% (Table 3). When compared with PPLs ≤20 mm, FDG‐PET for PPLs >20 mm showed a significantly higher diagnostic accuracy rate (*P* < 0.05) and a higher Youden's index.

**Table 4:** The SUV_max_ of FDG‐PET for PPLs grouped by lesion diameter and malignancy

	SUV_max_ (IQR)	*P*‐value
FDG‐PET		
≤20 mm	3.6 (1.7–8.6)	*P* = 0.03
>20 mm	7.3 (2.4–10.3)	
Benign PPLs	2.0 (1.5–3.1)	*P* < 0.01
Malignant PPLs	7.8 (2.8–10.3)	

IQR, interquartile range; PPLs, peripheral pulmonary lesions; SUV_max_, maximum standardized uptake value; FDG‐PET, fluorodeoxyglucose positron emission tomography.

### Diagnostic value of EBUS‐TBLB combined with FDG‐PET

When combining EBUS‐TBLB with FDG‐PET, the diagnostic accuracy rate, sensitivity, specificity, Youden' index, positive predictive value, and negative predictive value for PPLs were 86.8%, 90.2%, 73.3%, 63.5%, 93.2%, and 64.7%, respectively (Table 3). In the meantime, the diagnostic accuracy rate by combining EBUS‐TBLB with FDG‐PET was significantly higher than the single EBUS‐TBLB and FDG‐PET (*P* < 0.01 and *P* < 0.05, respectively). Furthermore, whether for PPLs >20 mm or ≤20 mm, the combined approach showed a significantly higher diagnostic accuracy rate than EBUS‐TBLB (*P* < 0.01 and *P* < 0.05, respectively), whereas no significant difference was found in comparison with FDG‐PET (Table 3). Additionally, the combined method was observed with a significantly higher diagnostic accuracy rate (*P* < 0.05) and a higher Youden's index for PPLs >20 mm than PPLs ≤20 mm. In total, 24 patients were identified by FDG‐PET as benign PPLs. Despite the negative FDG‐PET results, 13 patients were eventually confirmed to have malignant PPLs. Of these, seven (54%) could be identified by EBUS‐TBLB.

### Diagnostic accuracy rates for different clinical characteristics

The diagnostic accuracy rate of EBUS‐TBLB for negative bronchus sign was 33.3% (14.6%–57.0%), which was significantly lower than that (83.6%, 95% CI: 71.2%–92.2%), for positive bronchus sign (*P* < 0.001) (Table 5). Additionally, when using EBUS‐TBLB, PPLs within which the probe was positioned had a significantly higher diagnostic accuracy rate (80.8%, 95% CI: 67.5%–90.4%) than those where the probe was located adjacent to (45.8%, 95% CI: 25.6%–67.2%) (*P* = 0.002). We also observed that the diagnostic accuracy rate of PET for patients aged over 65 years was significantly higher than that for patients aged ≤65 years (*P* = 0.015).

**Table 5:** The diagnostic yields of the single and combined method for different characteristics

	Diagnostic yields (95% CI)		
Characteristics	PET	EBUS	PET+EBUS
Age (years)			
≤65	73.7 (56.9–86.6)*	68.4 (51.3–82.5)	84.2 (68.7–94.0)
>65	81.6 (65.7–92.3)	71.1 (54.1–84.6)	89.5 (75.2–97.1)
Bronchus sign			
Negative	76.2 (52.8–91.8)	33.3 (14.6–57.0)***	76.2 (52.8–91.8)
Positive	78.2 (65.0–88.2)	83.6 (71.2–92.2)	90.9 (80.0–97.0)
Probe within the PPLs			
No	75.0 (53.3–90.2)	45.8 (25.6–67.2)**	79.2 (57.8–92.9)
Yes	78.8 (65.3–88.9)	80.8 (67.5–90.4)	90.4 (79.0–96.8)

Note: *P < 0.05; **P < 0.01; ***P < 0.001.

CI, confidence interval; PPLs, peripheral pulmonary lesions.

## Discussion

To our knowledge, there are few studies evaluating the role of EBUS‐TBLB combined with FDG‐PET for diagnosing PPLs. Although Mizugaki *et al*.^9^ has demonstrated that a combination of EBUS‐GS‐TBLB with FDG‐PET could significantly increase the diagnostic yield for PPLs ≤30 mm, it is not clear whether the findings could be extended to EBUS‐TBLB without GS. Moreover, when the ultrasonic probe was covered by GS, its caliber was visibly enlarged, which was not in favor of the operation into the small angled branch of peripheral bronchi.^17^ Therefore, EBUS‐TBLB without GS is still adopted at some hospitals, especially in bronchoscopy units without GS, and it is important to determine the integrated value of EBUS‐TBLB with FDG‐PET in the assessment of PPLs.

When combining EBUS‐TBLB with FDG‐PET, an improved set of diagnostic indices (including diagnostic accuracy rate, negative predictive value and Youden's index) were observed. To be exact, the diagnostic accuracy rate, negative predictive value, and Youden's index were 86.8%, 64.7% and 63.5%, respectively. In general, 86.8% of diagnostic accuracy rate indicates that the probability of misdiagnosis made by the combined approach is 13.2%, and 64.7% of negative predictive value means a benign PPL diagnosed by the combined approach has 64.7% probability to be a true‐negative result. In addition, we also observed an increased Youden's index (sensitivity+specificity‐1), which meant the ability of identifying the true‐positive and true‐negative patients has been improved.^25^ In this sense, we have offered novel, essential evidence that EBUS‐TBLB in combination with FDG‐PET is particularly useful for diagnosing PPLs.

In this study, the diagnostic accuracy rate, sensitivity and specificity of EBUS‐TBLB were 69.7%, 62.3%, 100%, respectively, which is consistent with previous studies.^3^, ^10^, ^16^, ^17^, ^19^, ^26^ Besides, the diagnostic accuracy rate and sensitivity were found to be significantly higher in lesions >20 mm than lesions ≤20 mm, which has also been demonstrated by other studies.^10^, ^12^, ^16^, ^19^ This is mainly attributed to the technical problems, as the probe is less likely to be introduced into the correct location for a PPL ≤20 mm.

In the present study, we adopted the traditional cutoff SUV_max_ of 2.5, and PPLs with SUV_max_ >2.5 were considered positive.^4^, ^5^, ^6^, ^7^, ^8^ Consistent with previous studies,^7^, ^27^, ^28^ we found that FDG‐PET had a diagnostic accuracy rate of 77.6%, sensitivity of 78.7%, and specificity of 73.3%. The diagnostic accuracy rate of FDG‐PET was significantly higher for lesions >20 mm than lesions ≤20 mm, which was mainly owing to the partial volume effects.^29^, ^30^ As partial volume effects are strongly related to the size of tumor, smaller pulmonary nodules tend to have lower SUV_max_ than larger nodules, having been demonstrated by our findings where PPLs >20 mm displayed a significantly higher level of SUV_max_ than PPLs ≤20 mm. Additionally, our results are consistent with those of Khalaf *et al*. who found FDG‐PET had a significantly lower diagnostic value in the assessment of small‐size pulmonary nodules.^31^ Therefore, PPLs>20 mm are more likely to be accurately diagnosed by FDG‐PET.

Whether for EBUS‐TBLB or FDG‐PET, we observed a significant higher diagnostic accuracy rate for PPLs >20 mm than PPLs ≤20 mm. Allowing for this, it is understandable why the combined approach is more beneficial for the diagnosis of PPLs >20 mm, which is in agreement with the findings from Mizugaki *et al*.^9^ In addition, we found that positive bronchus sign and probe within the lesions were two important factors conducive to enhancing the diagnostic accuracy rate for EBUS‐TBLB, which was also demonstrated in other studies.^15^, ^21^, ^22^, ^23^, ^24^

Several limitations of this study should be noted. First, due to the single‐center, small sample size and retrospective nature of this study, the statistical power in our study may be lowered to some extent. Thus, a prospective, multicenter study with a larger sample size is encouraged to further confirm our findings. Second, since the accurate diagnosis of EBUS‐TBLB for PPLs is influenced by the operators' proficiency on this technique, and this study was conducted in the tertiary care center, the findings of this one‐center study may not be generalizable to the primary medical centers. Finally, as EBUS‐TBLB was performed using UM‐S20‐20R in the present study, our findings may not be applicable to EBUS‐TBLB using UM‐S20‐17R. In future, a new study combining UM‐S20‐17R with UM‐S20‐20R may be conducive to enhancing the diagnostic yields.

In conclusion, an integrated approach combining EBUS‐TBLB with FDG‐PET is particularly useful for diagnosing PPLs, with a diagnostic accuracy rate of 86.8%, sensitivity of 90.2%, specificity of 73.3%, Youden's index of 63.5%, positive predictive value of 93.2%, and negative predictive value of 64.7%. Meanwhile, the improved diagnostic yields were especially evident for PPLs >20 mm. Based on the results of EBUS‐TBLB and FDG‐PET, clinicians can make a preliminary judgment on the malignant or benign nature of PPLs, and decide on further surgical procedures or long‐term follow‐up.

## Disclosure

No authors report any conflict of interest.
